# The optimization method of wing ice shape regulation based on flight dynamics characteristics

**DOI:** 10.1038/s41598-022-22824-7

**Published:** 2022-10-29

**Authors:** Pengfei Dou, Zhe Li, Zehong Dong, Li-ke Xie

**Affiliations:** Aviation Engineering School, Air Force Engineering University, Xi’an, 710038 China

**Keywords:** Aerospace engineering, Plasma physics

## Abstract

Ice on aircraft wing changes the aircraft aerodynamic shape, and has negative effects on flight dynamic characteristics, seriously threatening flight safety. Plasma ice shape regulation is a new de-icing method. Plasma actuator produces an apparent thermal effect, which is designed to dissolve the continuous ice into intermittent ice pieces. How to achieve the optimal regulation ice shape to improve the flight dynamics characteristics under icing conditions is a technical problem restricting the application of this method. A simulation ice shape based on previous ice tunnel experiments and a scale model of swept wing were established. The aerodynamic parameters of no ice, full ice, and two regulation ice schemes were obtained based on wind tunnel. Six degrees of freedom flight dynamics model was established, and flight simulation had been carried out. As the analysis of trim characteristics, dynamic stability, and maneuverability, flight dynamics characteristics were better improved when the ratio of ice width to the mean aerodynamic chord was 0.15. The evaluation method of plasma ice shape regulation schemes was proposed. The proposed method, which can compare and optimize the arrangement of plasma actuators, realized the optimal regulation ice shape on the premise of balancing flight safety and energy consumption.

## Introduction

Ice on an aircraft wing changes the aerodynamic shape^1^, causing damage to the flight mechanical steering system^2^. In severe cases, it may even lead to losing control of the aircraft.^3^. In recent years, many serious flight accidents have been caused by icing^4^. Russian Saratov airliner An-148 got the wrong speed information for pilots due to icing on the airspeed tube, eventually crashed in 2018. General Aviation B-10GD Model Precipitation Enhancer crashed because of propeller icing in 2021 and five crew members were killed. Research showed that UAVs were more sensitive to ice conditions than that larger and faster manned aircraft and aircraft icing was one of the major hazards for airplane operation during takeoff and climbing missions at low temperature regions^5^,^6^. The aerodynamic characteristics of the aircraft are greatly affected by the ice accretion problems^7^. Innovative de-icing techniques are urgently needed.

At present, the two common de-icing techniques are heating and vibration^8^. Compared with vibration, heating de-icing system is mostly used for small components such as airspeed tubes, which has a slow response and low thermal efficiency^9^. Meanwhile, the low reliability of the vibration de-icing system would greatly damage the original aerodynamic shape of the aircraft^10^, and reduce the aerodynamic performance of the wing^11^. The de-icing technology of plasma has the advantages of low power consumption^12^, quick responses, and simple geometry^13^. Experiments have shown that its total energy efficiency is better under certain conditions^14^. Meng Xuanshi found that plasma de-icing method can improve the loss of aerodynamic characteristics^15^. Cem Kolbakir found that actuators in the stream wise laid out had a better de-icing effect than the actuators in the span wise layout. The results showed that plasma actuators in stream wise lay out not only prevented ice accretion near the airfoil leading edge but also allowed plasma-induced surface heating to further downward convection to prevent ice accretion near the airfoil trailing edge^16–18^. Rodrigues F. F found that de-icing technology based on plasma excitation had a better effect. The team studied the influence of the thickness of the exposed electrode on the performance of plasma actuators in the de-icing system, which found that the actuators with thicker exposed electrodes can induce higher average surface temperature, compared with the traditional actuator^19–21^. Isaac Ball found that the water film formed a virtual cathode on the surface of the plasma actuators, resulting in higher gas temperature and a larger heating area^22^. The ice shape regulation experiments with simulation ice were carried out. The results were consistent with the ice wind tunnel experiment. Compared with continuous ice, WU Yun found that plasma ice shape regulation can significantly improve aerodynamic performance, which can save about half of electric power and is very beneficial for application^23^.

Under certain conditions, the plasma actuator has a significant thermal effect. Its application has become a promising technique in the field of de-icing in the future^24^. However, due to the residual energy limit of the aircraft, ice-tolerant flight still exists^25^. The application of the plasma ice shape regulation method aims to improve the flight d1ynamic characteristics. The above scholars have studied the mechanism of plasma de-icing technology but did not carry out the flight dynamics characteristics. Obtaining the variation law of flight dynamics characteristics under plasma excitation is the premise of the application of this method. It is also the basis for optimizing the plasma ice shape regulation. Overall, this paper mainly studied: aerodynamic parameters under two kinds of regulation ice shapes based on wind tunnel experiments; six-degree-of-freedom flight dynamics model of the background aircraft is established; stimulation and comparison of the trim characteristic, dynamic stability, and maneuverability.

## Experiments and analysis of aerodynamic parameters

### The process of plasma ice shape regulation

**Figure 1:**
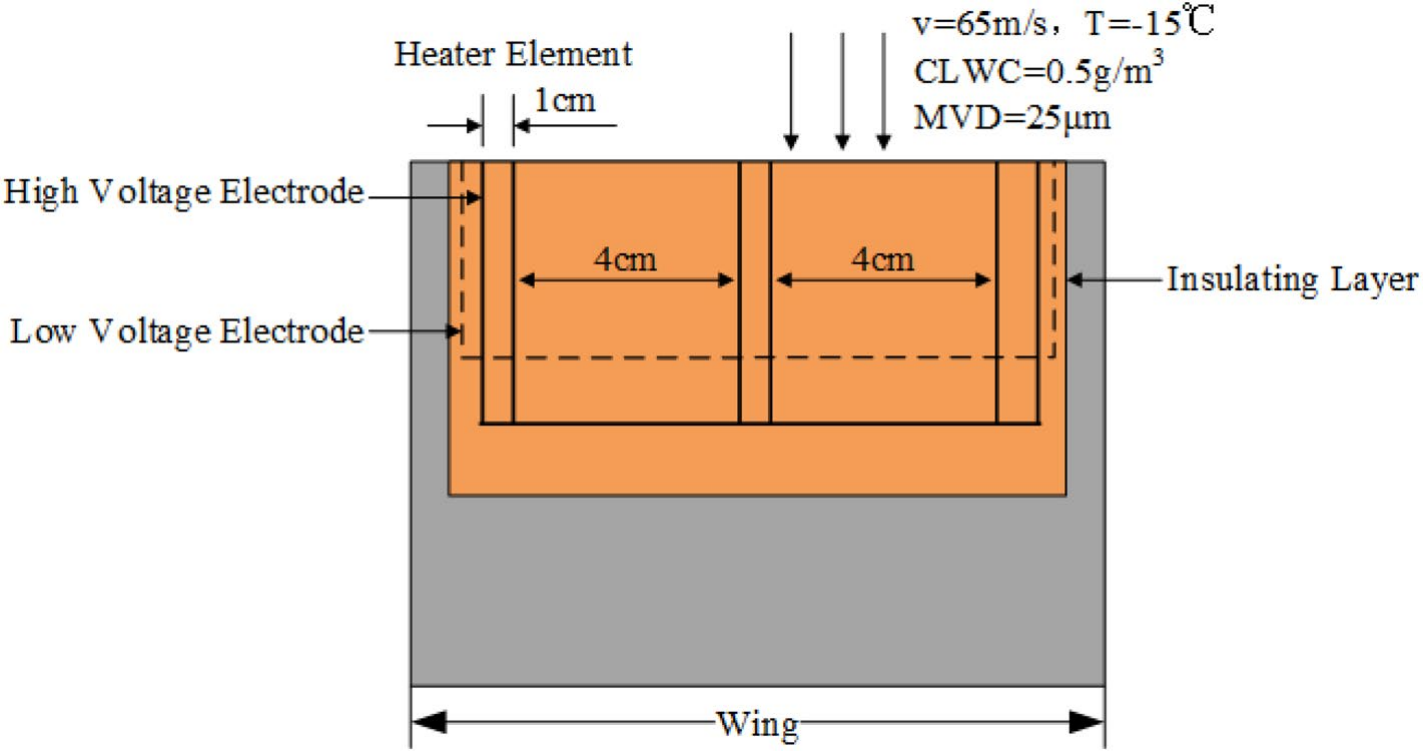
Diagrammatic drawing of plasma ice shape regulation actuator^23^.

**Figure 2:**
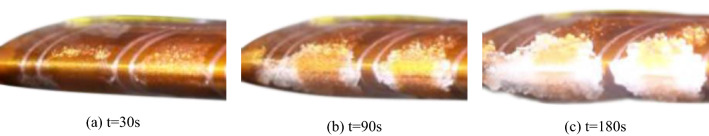
The process of plasma ice shape regulation at different moments^28^.

Plasma ice shape regulation mainly uses the thermal effect of the actuator to cut the continuous ice into intermittent ice. Figure 1 is a diagrammatic drawing of plasma ice shape regulation actuator, mainly composed of the heater and Non-Heater Elements. The width of a single heater element is 1 cm. According to the size of the scaled wing model, two heater elements are arranged at the wing leading edge. The spacing between each heater Element is 4 cm. It ensures that each Heater element does not affect the other, which can appear obvious wave leading edge^23^. The measurement of aerodynamic parameters of a wing with natural ice accretions is very difficult and expensive^26^. Icing wind tunnels are a critical engineering tool for simulating natural ice accretion on aircraft surfaces^27^. Our group has realized the ice shape regulation in the previous plasma de-icing experiment. Based on the ice wind tunnel, a preliminary exploratory experiment was conducted to study the plasma ice shape regulation under two meteorological conditions. As shown in Fig. 2, where the plasma actuator was arranged, ice was removed. Oppositely, where the plasma actuator was not arranged, ice accretion was serious. It showed that the continuous ice is cut into intermittent ice on the macro level. The width of the interval ice can be modulated by adjusting the arrangement of the plasma actuator. The research group called it ice shape regulation. According to the results of previous ice wind tunnel experiments, two-dimensional and three-dimensional data of ice shape regulation were collected based on three-dimensional scanning. The typical regulation ice shape is obtained^28^.

How to select the optimal ice shape regulation parameter which achieves the best de-icing effect is a new problem. Due to the high cost of the ice wind tunnel test^29^, a wind tunnel test with simulation ice based on a typical icing configuration was carried out. The aerodynamic parameters were analyzed under different conditions. In addition, due to the intense coupling relationship between flight parameters, the measurement of de-icing technology needs to be reflected in the flight dynamic characteristics. Aerodynamic parameters provide input for the simulation analysis of flight dynamic characteristics below.

### Background aircraft wing scale model and simulated ice shape

**Figure 3:**
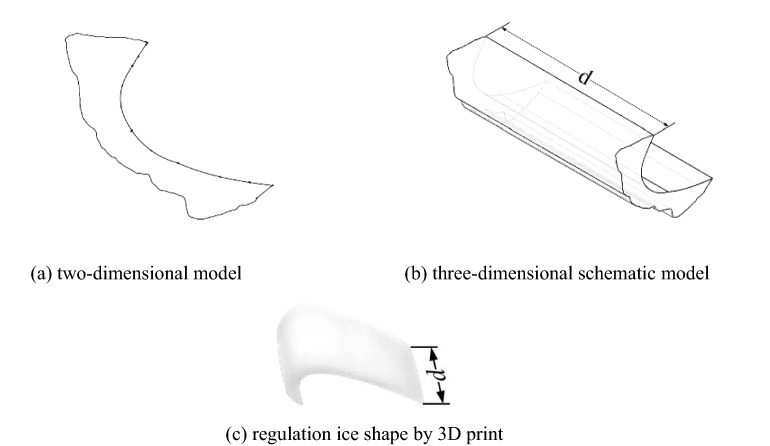
Background aircraft wing regulation ice shape.

**Figure 4:**
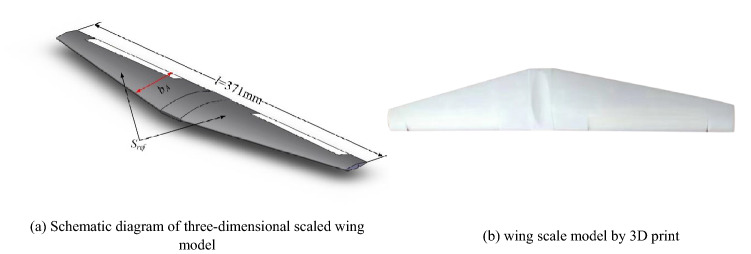
Background aircraft wing scaling model.

In reference^30^, the laws of swept wing icing were obtained by numerical simulation. The results showed icing thickness gradually increased from the wing root to the tip, which provides ice shape model support for the ice shape regulation experiment of the background aircraft wing scaling model. As shown in Fig. 3, combined with the icing laws of the swept wing and the typical regulation ice shape, the simulation regulation ice shape is established. Figure 3a is schematic diagram of two-dimensional regulation ice shape and Fig. 3b is three-dimensional. Figure 3c is a single regulation ice shape by 3D print. $d$ is the width of single regulation ice. The material used in 3D printing is nylon. The roughness of the model is 3.2, which is similar to those used in reference^23^. As shown in Fig. 4a, $l$ is span length, which is 371 mm. $S_{ref}$ is the area of the wing scale model, which is 41910mm^2^. $b_{A}$ is the mean aerodynamic chord, which is 110 mm. In Fig. 4b, the wing scale model by 3D printing is used in the experiment. The material used in 3D print is resin. The roughness of the model is 6.3, which is similar to those used in reference^23^.

**Figure 5:**
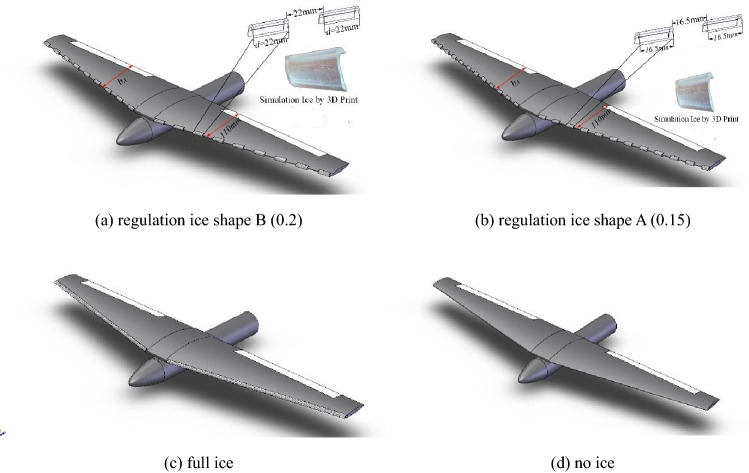
Different ice shape attached to wings.

As shown in Fig. 5, regulation ice A and B, full ice and no ice are arranged at the leading edge of the scaled wing model. $d$ is the width of single regulation ice. $b_{A}$ is the mean aerodynamic chord of the wing. This paper defined the width of single regulation ice as dimensionless ice size $c = d/b_{A}$. The regulation ice A was set as the dimensionless ice size $c = 0.15$. The regulation ice B was set as the dimensionless ice size $c = 0.2$. In the experiment, the simulation ice shapes were arranged in the leading edge of the wing model.

### Experimental equipment

**Figure 6:**
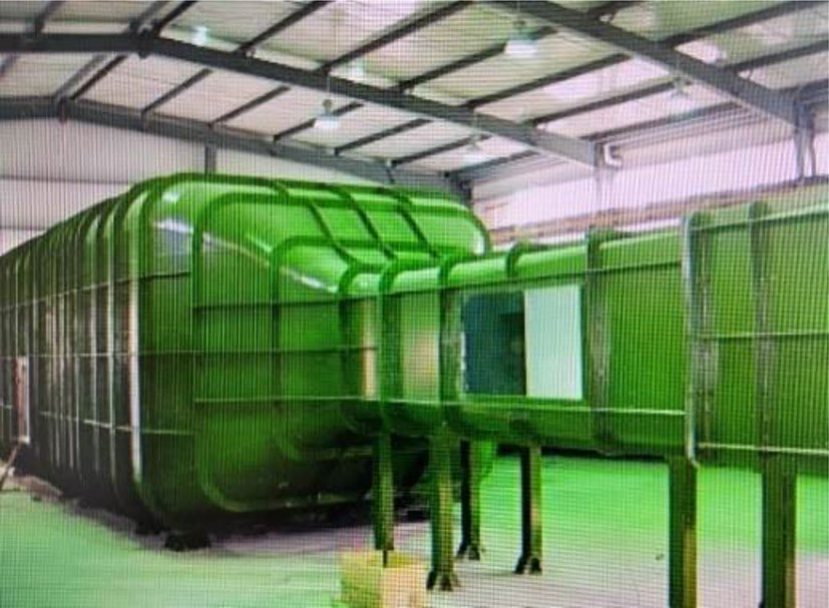
Low-speed backflow wind tunnel.

**Figure 7:**
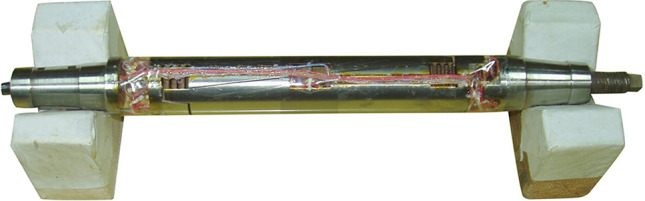
The schematic diagram of six-component for force-measuring balance.

Ice shape regulation experimental system includes a low-speed backflow wind tunnel, swept wing scale model, 3D simulation ice shape, and six-component force balance. Figure 6 shows the low-speed backflow wind tunnel used in the experiment, which consists of six parts. It is divided into an experimental section, diffuser section, transition section, stable section, contraction section, and dynamic section. The single reflux closed wind tunnel can produce a stable approaching flow. The length of the test section is 3 m. The test section is 1 m*1.2 m boxes. The wind speed is 5–75 m/s in the test section. Figure 7 shows the six-component aerodynamic force balance. The accuracy of the balance determines the credibility of the experimental results. Before the experiment, the static calibration frame was used for the static calibration of the balance. Since the model had no sideslip angle and roll angle in the experiment, the static calibration results in $x$ and $y$ directions were given. The results are shown in Table 1. The accuracy index of the balance has reached or exceeded the General specification for wind tunnel strain balances QJ 1884–1999. Where, the qualified index of balance precision is not less than 0.5% and the accuracy is not less than 0.7%.

**Table 1:** Experimental scheme of ice shape regulation.

Balance component	$x/{\text{kg}}$	$y/{\text{kg}}$	$m_{z} /{\text{kg}}*{\text{m}}$
Limit load	10	45	3
Precision	0.024	0.004	0.202
Accuracy	0.156	0.092	0.128

**Figure 8:**
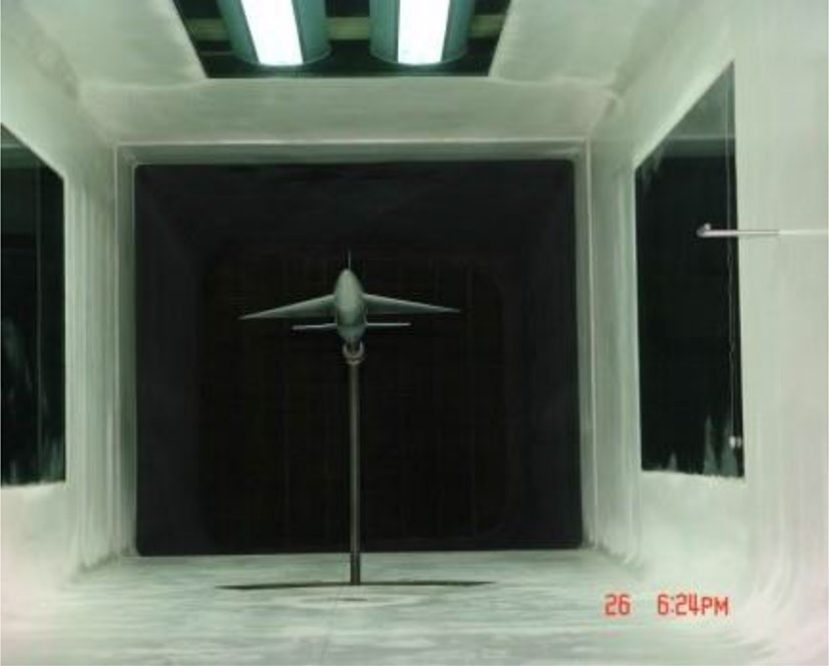
Diagram of ice shape regulation test.

As shown in Fig. 8, the incoming flow was 40 m/s and the air temperature was room temperature. The range of angle of attack (AOA) was from 0° to 22°. The lift coefficient, drag coefficient, and pitching moment coefficient of the scaled wing model were measured in the wind experiment test under the configuration of full ice, no ice, regulation ice A (0.15), and regulation ice B (0.2).

### Analysis of aerodynamic characteristics

**Figure 9:**
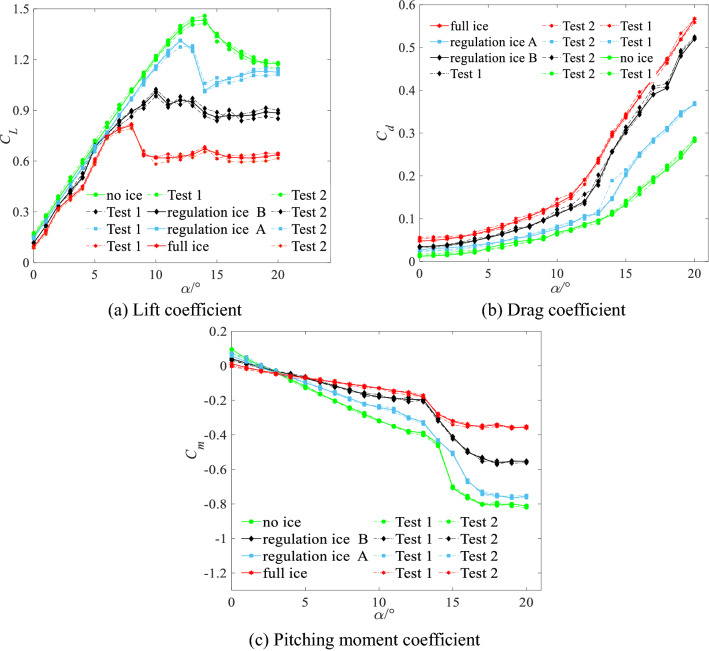
$C_{L}$, $C_{d}$, $C_{m}$ of background aircraft under four states.

The experimental schemes are shown in Table 2. To avoid the effects of uncertainties in the experiment, two wind tunnel force measurements were performed on the model in each state. As shown in Fig. 9, the lift coefficient $C_{L}$, drag coefficient $C_{d}$ and pitching moment coefficient $C_{m}$ under four states were measured. The dot dash is the first experimental result. The imaginary line is the second experimental result. The green solid line is the average of the aerodynamic parameters of the two experiments under no ice. The red is under full ice. The black is under regulation ice B. Blue is under regulation ice A. Observing the curves of the two experiments, it is found that the aerodynamic characteristic curves basically coincide in the linear region and was slightly different in the nonlinear region.

1 $$\delta_{i} = \frac{{\sqrt {\frac{{\left( {x_{i} - \overline{x}} \right)^{2} }}{n - 1}} }}{{\overline{x}}}$$

**Table 2:** Experimental scheme of ice shape regulation.

Ice state	Dimensionless ice size c
No ice	0
Regulation ice A	0.15
Regulation ice B	0.2
Full ice	4

The reproducibility parameters $\delta_{i}$ were calculated by Eq. (1) under different angles of attack (AOA) in each state. Where, $x_{i}$ is the measured values. $\overline{x}$ is the average of two experimental measurements experimental^31^. As shown in Table 3, the results show that the average of reproducibility parameters ($\overline{\delta }$) at all angles of attack does not exceed 5%, with good repeatability.

**Table 3:** Calculation results of repeatability parameters ($\overline{\delta }$).

Ice state	Lift coefficient $\overline{\delta }$	Drag coefficient $\overline{\delta }$	Pitching moment coefficient $\overline{\delta }$
No ice	0.0475	0.0401	0.0112
Regulation ice A (0.15)	0.0354	0.0482	0.0421
Regulation ice B (0.2)	0.0351	0.0346	0.0257
Full ice	0.0417	0.0384	0.0286

As shown in Fig. 9, the maximum lift coefficient of the full ice state ($C_{LMax4}$) reduces by 53.4% and the stall angle reduces by 42.9% compared with the no ice state. This is because ice accretion changes the aerodynamic shape of the wing surface. The flow field characteristics become worse, and the aerodynamic performance gradually deviates from the stable state. By the method of ice shape regulation and compared with the full ice state, the maximum $C_{L}$ under regulation ice B and A state ($C_{LMax3}$, $C_{LMax2}$) increases by 31.4% and 38.5%, and the stall Angle of Attack (AOA) increases by 25.1% and 51.2%.

The drag coefficient $C_{d}$ changes slightly under four states from 0° to 10°, which indicates that the repeatability of the wind tunnel force measurement system is better. The drag coefficient $C_{d}$ increases rapidly within the range of large AOA. Taking $\alpha = 12^\circ$ as an example, the drag coefficient $C_{d}$ increases by 104.5% under the full ice state and compared with no ice. Compared with the full ice state, the increasing extent of drag coefficient $C_{d}$ reduces by 72.3% and 17.8% under regulation ice B and A states. Between them, the decreasing extent of drag coefficient $C_{d}$ under regulation ice A state is higher than B state. Under the regulation ice A state, it better improves the phenomenon that the drag increases significantly.

The pitching moment coefficient $C_{m}$ is not significantly different from the no ice state within the range of small AOA. The pitching moment coefficient $C_{m}$ decreases sharply within the range of large AOA. Under the full ice state and compared with the no ice state, the pitching moment coefficient $C_{m}$ reduces by 56.2% at the stall AOA. Under regulation ice B and A state, the decreasing extent of the pitching moment coefficient $C_{m}$ increases by 11.5% and 51.2% compared with the full ice state. The aerodynamic characteristics of background aircraft in pitching direction are better improved under regulation ice A state.

**Figure 10:**
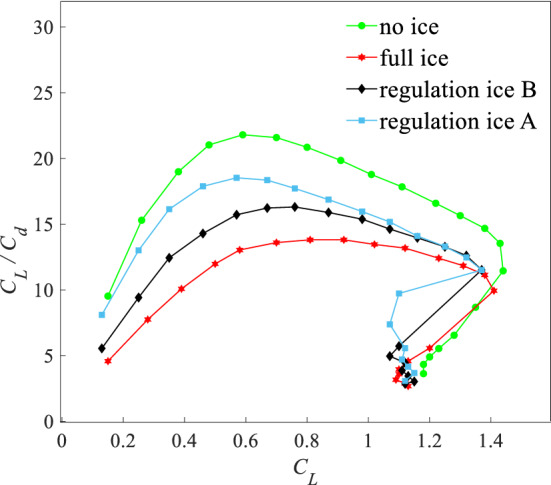
Lift-drag characteristic curves under four states.

**Figure 11:**
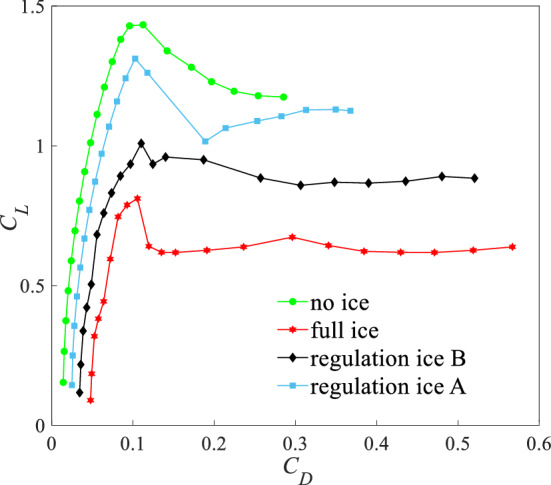
Polar curves under four states.

The lift-resistance ratio is $K = C_{L} /C_{d}$. Figure 10 is the lift-resistance characteristic curves under four states. Compared with the no ice state, the maximum lift-drag ratio $K$ reduces by 62.9% under the full ice state. Compared with the full ice state, the maximum $K$ increases by 12.5% and 38.5% under the regulation ice B and A states. Figure 11 is the polar curves under four states. The slope of every point represents the lift-drag ratio $K$ in the polar curve. Under the regulation ice A state and compared with B state, the lift coefficient $C_{L}$ increases faster and the drag coefficient $C_{d}$ increases slowly from zero lift AOA to favorable AOA. When exceeds critical AOA, the results show that an increasing extent of lift-drag ratio ($K$) is more obvious under regulation ice A state. The airfoil aerodynamic efficiency is improved.

This section analyses lift-resistance characteristics and pitching moment characteristics under four states. It can show that the method of ice shape regulation can improve the aerodynamic efficiency under the full ice state and slow down the damage to the aerodynamic characteristics under the ice accretion state. When the scheme of regulation ice A (0.15) is selected, the aerodynamic characteristics are better improved compared with the scheme of regulation ice B (0.2).

## Dynamics modeling of background aircraft under ice condition

**Figure 12:**
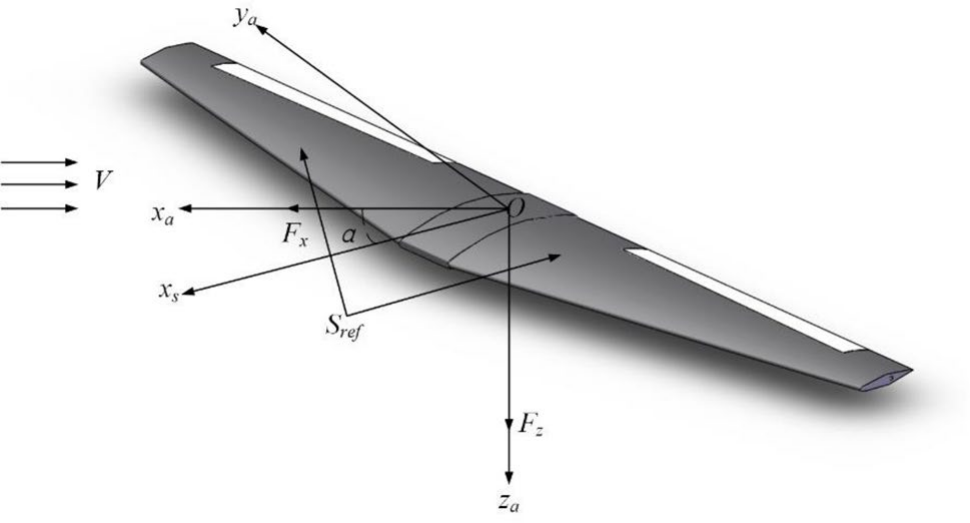
Diagram of American coordinate system and scale model parameter.

Figure 12 is the American coordinate system used in the flight dynamics simulation and the schematic diagram of the geometric parameters of the model and surface force. Here: The origin $O$ is located in the center of mass. $x_{a}$ is along flight velocity direction. $z_{a}$ is in the plane symmetry plane, which is perpendicular to the direction of $x_{a}$ below. $y_{a}$ is perpendicular to the plane where $x_{a}$ and $z_{a}$ are located to the right.

Taking the longitudinal Dynamics Characteristic as an example, the mathematical model of background aircraft is constructed as shown in Eqs. (2)–(5)^32^:

2 $$\mathop V\limits^{.} = \frac{1}{{\text{m}}}\left( {F{}_{x}\cos {\upalpha + }F{}_{z}\sin \upalpha } \right)$$

3 $$\mathop \upalpha \limits^{.} = \frac{1}{{{\text{m}}V}}\left( { - F_{x} \sin \upalpha + F_{z} \cos \upalpha } \right) + q$$

4 $$\dot{\theta } = q$$

5 $$\mathop q\limits^{.} = \frac{{M_{y} }}{{I_{yy} }}$$

Here: $m$ is the aircraft quality. $V$ is flight velocity. $\alpha$ is the AOA. $\theta$ is pitch angle. $q$ is pitch angle rate. $I_{yy}$ is the longitudinal moment of inertia. $M_{y}$ is the pitching moment. $F_{x}$ and $F_{z}$ are the component of the resultant force on the body axis in the *x* and *z* direction. In Eqs. (6)–(8), $F_{x}$, $F_{z}$ and $M_{{\text{y}}}$ are as follows:

6 $$F_{x} = \overline{q}S_{ref} \left[ {C_{x} \left( \alpha \right) + C_{x} \left( {\alpha ,\delta_{e} } \right) + C_{x} \left( {\alpha ,\hat{q}} \right)} \right] + 2T_{X} \left( {\delta_{th} } \right) - mgsin\theta$$

7 $$\begin{gathered} \hfill \\ F_{z} = \overline{q}S_{ref} \left[ {C_{z} \left( \alpha \right) + C_{z} \left( {\alpha ,\delta_{e} } \right) + C_{z} \left( {\alpha ,\hat{q}} \right)} \right] + 2T_{Z} \left( {\delta_{th} } \right) - mg\cos \theta \hfill \\ \end{gathered}$$

8 $$M_{y} = \overline{q}S_{ref} \overline{c}\left[ {C_{m} \left( \alpha \right) + C_{m} \left( {\alpha ,\delta_{e} } \right) + C_{m} \left( {\alpha ,\hat{q}} \right)} \right] + 2\Delta {\text{Z}}_{ENG} T_{X} \left( {\delta_{th} } \right)$$

Here: $\overline{q}$ is dynamic pressure. $S_{ref}$ is the area of wings. $C_{x}$, $C_{{\text{z}}}$, $C_{m}$ is force and moment coefficient respectively. $T_{x}$ and $T_{y}$ is projection of engine thrust in the axis and direction of the body. $g$ is gravity acceleration. $\delta_{e}$ is the elevator angle. $\delta_{th}$ is throttle position. $\Delta Z_{ENG}$ is projection of the relative position vector of the engine moment centroid in the axial direction of the body.

In Eq. (9), the pitch-holding control configuration is adopted in the study, the specific expression is as follows:

9 $$\delta_{e} = K_{\uptheta } \left( {\uptheta _{c} { - \theta }} \right){ + \text{K}}_{\alpha }\Delta \alpha { + \text{K}}_{q} q$$

where $K_{\theta }$, $K_{\alpha }$, $K_{q}$ are feedback gain of flight state $\theta$, $\alpha$ and $q$. The values are invoked by cubic spline interpolation at different state points. The control parameters are:

10 $$K_{\uptheta } = 2$$

11 $$K_{\alpha } = 1.5$$

12 $$\text{K}_{q} = 0.2$$

## Analysis of dynamic characteristics

### Analysis of trim characteristic

In order to ensure the stable level flight of the aircraft, the force and torque acting on the aircraft must be balanced. The process of a stable balance of the aircraft is called balancing. At present, there are two ways to maintain the balancing state of the aircraft in horizontal flight. To make longitudinal torque zero, one is that pilot manipulates the control stick changing the size and direction of the pitching torque. The other is using trim tab, and the pilot operates without a control stick^33^. When the aircraft speed changes, the aircraft is required to balance again. The longitudinal trim characteristics are as an example. Several parameters are selected in the balancing process, such as the Angle of Attack (AOA), the elevator angle, and the throttle equivalent. The elevator angle for balancing refers to the angle of the elevator required to maintain the aircraft balance. The AOA for balancing refers to the angle required to maintain the pitch balance. The throttle equivalent for balancing refers to the thrust equivalent provided by the engine during balancing, which represents the position of the throttle rod. This section calculated the AOA, elevator angle, and throttle equivalent for balancing under four states of no ice, full ice, regulation ice A and B. And the trim characteristics were compared and analyzed under four states.

In order to characterize the icing severity under different regulation ice shapes, the concept of icing factor $\eta_{iced}$ is introduced. Professor Bragg proposed a universal icing impact model based on estimates. In Eq. (13), the aerodynamic derivative relationship before and after icing is as follows^34^:

13 $$C_{(A)iced} = (1 + \eta_{iced} k_{CA} )C_{(A)}$$

where $C_{(A)}$ is any aerodynamic derivative. $C_{(A)iced}$ is the corresponding aerodynamic derivative under icing state.$K_{CA}$ is the ice factor, usually obtained by flight simulation. For the aircraft, $\eta_{iced}$ is a constant. As a meteorological factor, the more serious aircraft icing is, the greater the icing factor has, and $\eta_{iced}$ is usually 0–0.4^35^.

**Figure 13:**
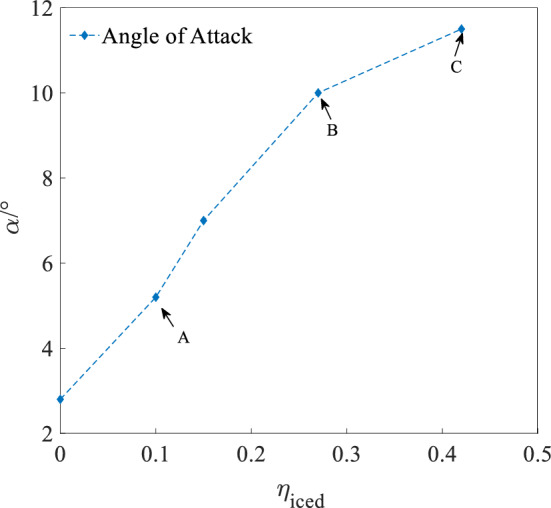
Balancing value of angle of attack.

**Figure 14:**
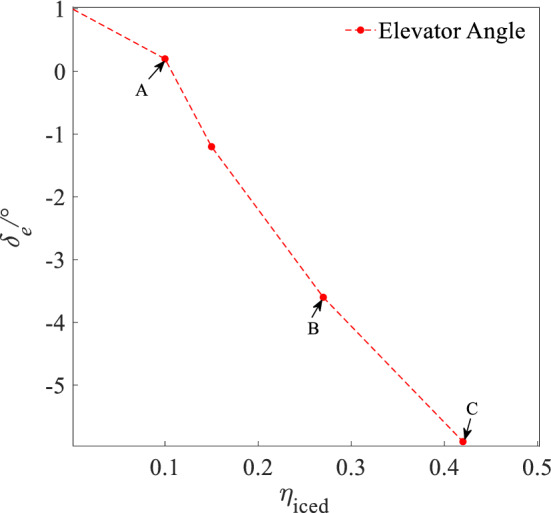
Balancing value of elevator angle.

**Figure 15:**
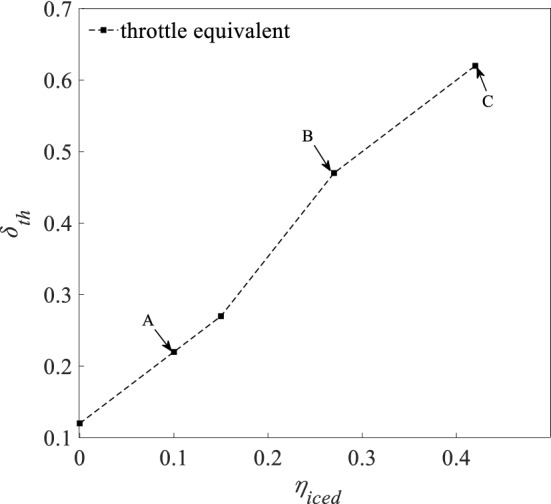
Balancing value of throttle equivalent.

Figures 13, 14 and 15 shows AOA ($\alpha$), elevator angle ($\delta_{e}$), and throttle equivalent ($\delta_{th}$) for balancing under four states of regulation ice A and B, no ice and full ice state. In Figs. 13, 14 and 15, points A, B, and C separately expresses the state of regulation ice A and B and full ice.

Compared with the no ice state, the AOA $\alpha$ for balancing is larger under the full ice state. With the increase of icing factor $\eta_{{{\text{iced}}}}$, the AOA $\alpha$ for balancing increases. This is because that icing leads to the decrease of the lift coefficient. In order to maintain stable level flight, it is necessary to increase AOA $\alpha$ to compensate loss of the lift. The increase of AOA $\alpha$ needs to increase the elevator angle $\delta_{e}$ to maintain the longitudinal balance. Because elevator efficiency reduces under the ice state, the elevator angle $\delta_{e}$ for balancing is also larger than that under the no ice state. The drag coefficient $C_{d}$ of the aircraft increases under the icing state, so it requires the engine to provide greater thrust. The throttle equivalent $\delta_{th}$ for balancing is also increased by the increase of $\eta_{iced}$. Under regulation ice shape A and B, the AOA $\alpha$ for balancing reduces by 10% and 45.5%. The elevator angle $\delta_{e}$ for balancing reduces by 33.3% and 94.5%. The throttle equivalent $\delta_{th}$ for balancing reduces by 23.6% and 35.2%, compared with the full ice state.

The results show that the AOA, elevator angle, and throttle equivalent for balancing is reduced by the decrease of dimensionless ice shape value. Especially, the scheme of the regulation ice A (0.15) is selected, which can better improve the longitudinal trim characteristics of the aircraft. The efficiency of balancing is enhanced.

### Analysis of dynamic stability characteristic

**Figure 16:**
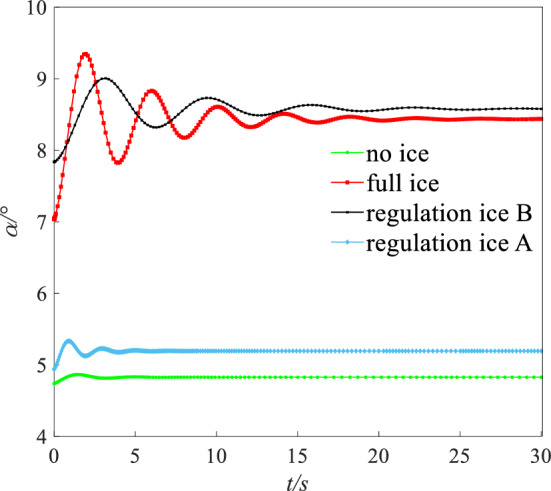
Comparison of angle of attack dynamic stability.

**Figure 17:**
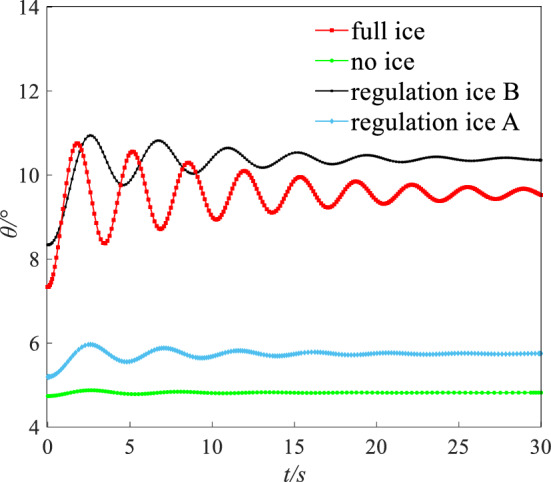
Comparison of pitching angle dynamic stability.

**Figure 18:**
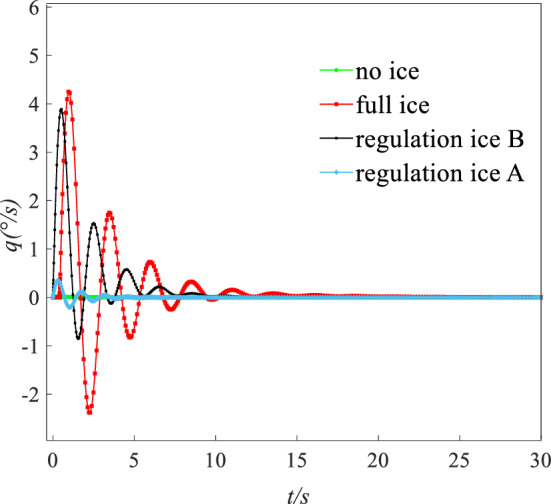
Comparison of pitching angular rate dynamic stability.

The flight height was set to 400 m. The speed was set to 0.21 Ma. Under this state (height: 400 m, speed: 0.21 Ma), the AOA $\alpha$, elevator angle $\delta_{e}$, and throttle equivalent $\delta_{th}$ for balancing was set as the initial value. The initial values of $\alpha$, $\delta_{e}$, and $\delta_{th}$ for simulation are shown in Table 4. A disturbance with $\Delta \alpha = 1.5^\circ$ was given. Simulating 30 s, the dynamic response of pitching angle ($\theta$), AOA ($\alpha$), and pitching angular rate ($q$) is obtained as shown in Figs. 16, 17 and 18.

**Table 4:** The initial values for dynamic simulation.

Ice state	Angle of attack $\alpha /{^\circ }$	Elevator angle $\delta_{e} /{^\circ }$	Throttle equivalent $\delta_{th}$ (%)
No ice	3.52	0.96	13
Regulation ice A (0.15)	4.59	0	22
Regulation ice B (0.2)	8.52	− 3.72	46
Full ice	10.1	− 5.96	61

Under the disturbance with $\Delta \alpha = 1.5^\circ$, the flight state parameters have a dynamic oscillation process. Under the full ice state, the AOA $\alpha$ increases sharply. The initial response peak of AOA is 9.5°, which is close to the stall AOA under the full ice state. If the pilot mishandles slightly, which is easy to cause the aircraft stall and flight accident. In addition, AOA $\alpha$, pitching angular rate $q$, and pitching angle $\theta$ in the short period has sharp oscillation and the oscillation amplitude is larger under the full ice state. It may lead to the uncontrollable situation of aircraft.

However, compared with the full ice state, the peak value of the initial AOA $\alpha$ reduces by 11.2% and 31.2% under regulation ice shape B and A. The AOA $\alpha$ can converge back to steady state faster. The pitching angular rate $q$ is the main influence factor of short period mode. Under full ice state, pitching angular rate $q$ converges back to the steady state at 10 s. Under regulation ice shape A and B, pitching angular rate $q$ converges back to the steady state at 5 s and 8 s, which reduces by 50% and 20% compared with the full ice state. When the disturbance disappears, the pitching angle $\theta$ is the main influence factor of long period mode. Under the full ice state, the pitching angle $\theta$ has sharp oscillation and converges back to the stable state at 25 s. Under regulation ice shape A and B, it gradually stabilizes at 5 s and 10 s.

Under disturbance, the results show that the oscillation period and amplitude of the short/long period mode parameters are significantly reduced and can converge back to a stable state faster by ice shape regulation. Especially, the scheme of the regulation ice A (0.15) is selected, the longitudinal dynamic stability characteristics are better improved compared with the full ice state.

### Analysis of maneuverability

**Figure 19:**
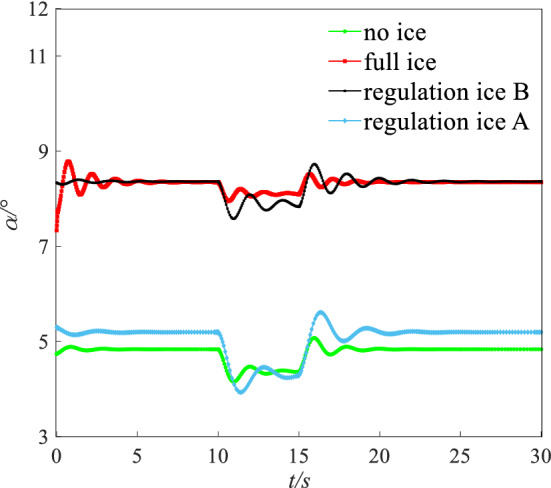
Dynamic response of angle of attack.

**Figure 20:**
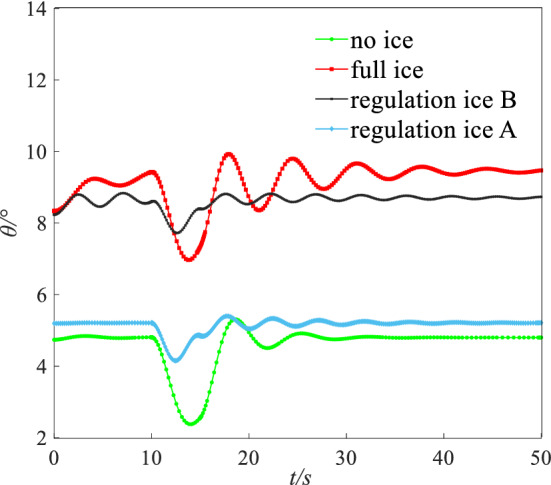
Dynamic response of pitching angle.

**Figure 21:**
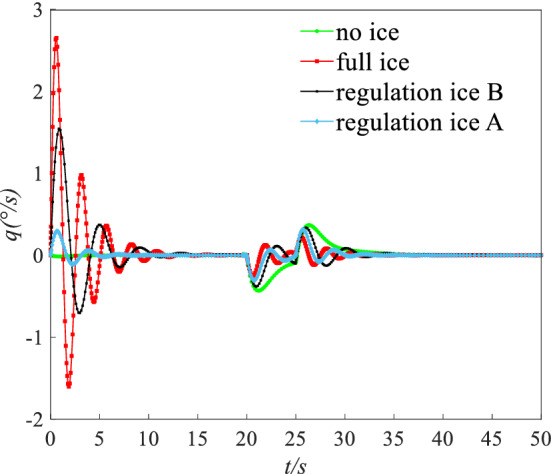
Dynamic response of pitching angular rate.

A step signal with width of 5 s and amplitude of 2° was imposed on the elevator. The dynamic response including pitching angle ($\theta$), AOA ($\alpha$), and pitching angular rate ($q$) under four states are shown in the Figs. 19, 20 and 21.

Under a step input to the elevator, the results show that the state parameters occur typical dynamic oscillation process under the full ice and no ice states. Under no ice state, parameters of short period mode including AOA $\alpha$ and pitching angular rate $q$ rapidly show a convergence trend. Because the damping ratio becomes smaller after icing, the flight state parameters tend to diverge. The dynamic response of long and short period parameters gets the process of nonlinear oscillation under the full ice state. The mode parameters slowly converge back to the equilibrium point. The AOA $\alpha$ that maintains level flight is larger compared with the no ice state. This is mainly because the lift coefficient reduced under icing state. The pitching angle $\theta$ of long period mode parameter has larger oscillation period and amplitude.

By ice shape regulation, oscillation amplitude and period of long/short model parameters decrease and converge to an equilibrium point faster compared with the full ice state. Under the regulation ice A state, short period mode parameters of AOA $\alpha$ and pitching angular rate $q$ converge back to the equilibrium point at the 3 s and 4 s. Under the regulation ice B state, the short period mode parameters converge back to the equilibrium point at the 5 s and 8 s. The long period mode parameter of pitching angle $\theta$ converges back to the equilibrium point at the 3 s and 5 s under the regulation ice A and B. The main reason is that the mode damping ratio of long and short periods increases by ice shape regulation and the oscillation period decreases. Especially, when the scheme of the regulation ice A (0.15) is selected, maneuvering efficiency of elevator has been enhanced and the mode parameters of aircraft respond faster. The ability to maintain balance increases and the flight quality is improved.

## Conclusions

Based on aerodynamic characteristics, flight dynamic characteristics, and flight quality evaluation, the researchers proposed a comparison method of schemes of plasma ice shape regulation. Results show that the aerodynamic and flight dynamics characteristics are better improved when the scheme in which the ratio of ice width to mean aerodynamic chord is 0.15 (regulation ice A).

1. By ice shape regulation, the lift and pitching moment coefficient increases and the drag coefficient decreased compared with the full ice state. Especially, the maximum lift coefficient configuration increased by 38.5% and the stall AOA increased by 4 ${^\circ }$ under regulation ice A state. The aerodynamic characteristics of the aircraft were better improved.
2. With the increase of icing factor $\eta_{{{\text{iced}}}}$, AOA, elevator angle, and throttle equivalent for balancing increased. The parameters for balancing were decreased by the ice shape regulation, which indicates the maneuverability is improved. Especially, AOA $\alpha$ for balancing reduced by 45.5%. Elevator angle $\delta_{e}$ reduced by 94.5% and throttle equivalent $\delta_{th}$ reduced by 35.2% under the regulation ice A state.
3. A disturbance with $\Delta \alpha = 1.5^\circ$ was given. Under full ice state, the AOA $\alpha$ increased sharply and the initial response peak was 9.5 ${^\circ }$. The peak of the initial AOA $\alpha$ decreased by 11% and 31.2% under the regulation ice shape B and A state. A step signal was applied to the elevator. Under the full ice state, the long and short period model parameters slowly converged back to the equilibrium point. The mode parameters can converge back to the equilibrium point in a shorter time by ice shape regulation. Especially, the long and short period model characteristics were better improved and the flight quality was improved when a scheme of the regulation ice A was selected.

In the future, schemes of different regulation ice shapes will be studied under different airfoils and different icing conditions. More detailed work will be studied on the laws of ice shape regulation schemes with aerodynamic and flight dynamics characteristics and flight quality. The essay is supposed to offer technical support for plasma ice shape regulation technology and reference for disaster mechanism of icing.

## Data Availability

All data generated or analyzed during this study are included in this published article and the datasets used during the current study available from the corresponding author on reasonable request.
